# Reverse Shoulder Arthroplasty for the Treatment of 3 and 4- Part Fractures of the Humeral Head in the Elderly

**DOI:** 10.2174/1874325001711010108

**Published:** 2017-02-28

**Authors:** Ioannis Gigis, Alexandros Nenopoulos, Dimitrios Giannekas, Roderich Heikenfeld, Theodoros Beslikas, Ippokratis Hatzokos

**Affiliations:** 1 2^nd^ Orthopedic Department, Aristotle University of Thessaloniki, “G. Gennimatas” General Hospital Thessaloniki, Thessaloniki, Greece; 2 Center for Shoulder, Elbow and Hand Surgery, Center for Orthopaedics and Traumatology of the St. Elisabeth Group - Catholic Hospitals Rhein-Ruhr, St. Anna Hospital Herne, Marienhospital Herne University Hopsital, Marienhospital Witten, Germany

**Keywords:** Elderly, proximal humerus fractures, Reverse shoulder prosthesis, Fragility, Shoulder hemiarthroplasty, Internal fixation, Tuberosities fracture

## Abstract

**Background:**

Proximal humeral fractures in elderly patients present with severe comminution and osteoporotic bone quality.

Reverse shoulder arthroplasty has lately been proven beneficial in treating patients with complex proximal humeral fractures. The above technique is recommended and has better results in elderly than in younger individuals.

**Methods:**

We performed a literature search in the databases Pubmed, Medline, EMBASE and Cochrane Library for published articles between 1970 and 2016 using the terms: proximal humerus fractures and reverse shoulder arthroplasty.

**Results:**

Significant benefits with the use of reverse prosthesis, especially in patients older than 70 years with a proximal humeral fracture, include reduced rehabilitation time as well as conservation of a fixed fulcrum for deltoid action in case of rotator cuff failure.

Compared with hemiarthroplasty and internal fixation, reverse prosthesis may be particularly useful and give superior outcomes in older patients, due to comminuted fractures in osteopenic bones.

However, significant disadvantages of this technique are potential complications and a demanding learning curve.Therefore, trained surgeons should follow specific indications when applying the particular treatment of proximal humeral fractures and be familiar with the surgical technique.

**Conclusion:**

Although long-term results and randomized studies for reverse prosthesis are lacking, short and mid- term outcomes have given promising results encouraging more shoulder surgeons to use this type of prosthesis in proximal humeral fractures.

## INTRODUCTION

Proximal humeral fractures are frequently seen in adults and account for about 5% of all fractures with an increasing rate in elderly population [1, 2].

The majority of proximal humeral fractures do not require surgical operation and can have excellent outcomes following conservative treatment [3-5].

The decision for surgery depends on patient comorbidities, functional demands, bone quality and surgeon experience [6-9].Treatment with open reduction and internal fixation or hemiarthroplasty is not recommended for fractures with poor bone quality and high grade of comminution [10]

Reverse shoulder arthroplasty (RSA) is an alternative treatment for complex 3 and for 4part proximal humeral fractures in elderly population with encouraging early results and patient outcomes [11-14].

## AVAILABLE TREATMENTS OF PROXIMAL HUMERAL FRACTURES

### Conservative Treatment

Nonoperative management involves systematic analgesia and sling immobilization. Generally, it is best preserved for non-displaced fractures of the proximal humerus or for patients medically unsuitable for surgery.

When compared to prolonged immobilization, early physiotherapy starting two weeks after injury was found to have better outcomes [15, 16]. Three and 4-part fractures have been treated nonoperatively but with poor results. Predicting the outcome of 3 or 4-part fractures treated conservatively is difficult, and a patient’s decision not to undergo surgery may have its own untoward consequences. Complications encountered with conservative treatment of these fractures include malunion and nonunion, subacromial impingement, avascular necrosis of the humeral head, stiffness secondary to osteoarthritis rotator cuff deficiency and shoulder pain. Malunions of the proximal humerus can cause serious restriction in external rotation and abduction from a mal-positioned greater tuberosity fragment. Osteonecrosis can also cause humeral head collapse, leading to degenerative changes of the glenohumeral joint, although some patients with osteonecrosis may still have a satisfactory outcome [17] Recent studies [4, 18] noted good functional results with nonoperative management. However, the majority of the fractures included were non-displaced or minimally displaced. If the humerus heals in a severely malunited position, the shoulder may not be amenable to future reconstruction with a standard total shoulder arthroplasty making RSA the necessitate treatment for these conditions. Surgery should at least be considered in active and healthy patients with 3- and 4-part fractures because it can potentially restore anatomy and consequently improve function.

### Open Reduction and Internal Fixation with Locking Plates

Younger adults with adequate bone quality can benefit more from open reduction and internal fixation with the use of locking plate osteosynthesis. The technique can have positive outcomes especially in large and solid tuberosity fracture fragments.

**Fig. (1):**
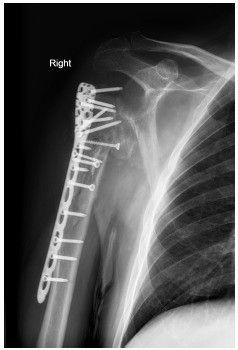
Avascular necrosis and screw penetration of humeral head following plate osteosynthesis of a 4-part proximal humeral fracture.

After the introduction of locking plates indications for this type of treatment were increased but recent studies showed a high rate of complications such as fracture displacement, screw cut out up to 57%, intra-articular migration of screws and avascular necrosis of humeral head up to 55% [19, 20] (Fig. **1**).

Two requirements for avoiding the risk of fixation failure are the anatomical reduction and the restoration of the neck-shaft angle.

**Fig. (2):**
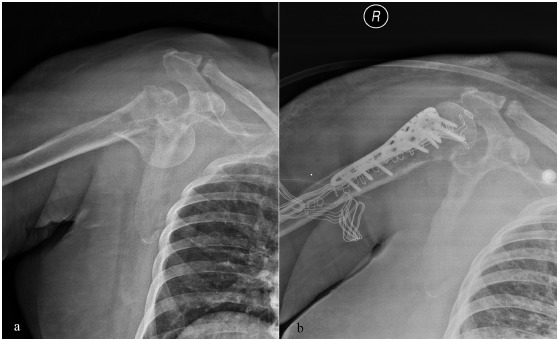
Proximal humeral head fracture with extension to the shaft (**a**) treated with plate osteosynthesis (**b**).

On the other hand, a number of studies support the use of medial support screws as a main method of maintaining reduction of unstable three- and four-part fractures (Fig. **2**). A study evaluating the outcomes of complex proximal humeral fractures in the elderly [21] resulted in a 51% early failure rate and a 26% need for reoperation in the treatment of 82 shoulders with osteosynthesis. Authors concluded that osteosynthesis of these fractures with locking plates had lower but significant rate of malunions and malpositioning of fracture fragments preventing patients from acceptable outcomes.

### Hemiarthroplasty

**Fig. (3):**
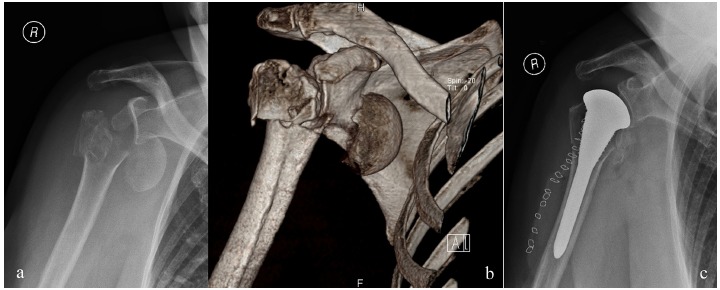
X-ray (**a**) and 3D reconstruction CT (**b**) of a 4-part humeral head fracture treated with hemiarthroplasty (**c**).

Neer [22] was the first to introduce hemiarthroplasty as a treatment alternative. It offers a good solution for comminuted 3 and 4-part fractures of proximal humerus (Fig. **3**) providing pain relief and outcomes ranging between excellent and poor. Key point for hemiarthroplasty success is tuberosities healing [23] which offers satisfactory outcomes and good range of motion. In case of tuberosity resorption, nonunion, or malunion poor outcomes are often seen. Long-term follow-up of such fractures treated with hemiarthroplasty verify pain relief but vary in functional outcomes [24, 25]. It appears that better results can be obtained in younger patients and patients with less comminuted tuberosities. Improper retroversion, poor tuberosity positioning, and excessive prosthetic height have been implicated as factors associated with poor functional results. Complications include aseptic loosening, dislocation, infection and reflex sympathetic dystrophy. Other type of complications may include subacromial impingement, intraoperative or periprosthetic fractures, rotator cuff dysfunction secondary to tuberosity displacement and resorption and heterotopic ossification [26]. In combination with older age, implant malpositioning, increasing degree of tuberosity displacement, or even a persistent neurological deficit, these can lead to poor outcomes, creating the need for early re-operation and use of hemiarthroplasty for salvage of previous failed procedures [27, 28]. Failures due to improperly functioning rotator cuff often requiring additional surgery according to some long-term follow-up studies. Therefore, hemiarthroplasty offers significant pain relief but variable range of motion and outcome that often depends on tuberosity malposition and nonunion. When hemiarthroplasty is used, proper implant position and tuberosity reduction and fixation are critical.

### Reverse Shoulder Arthroplasty

A technique by Paul Grammont called Reverse Shoulder Arthroplasty (RSA) was initially proposed for the treatment of glenohumeral arthritis with rotator cuff arthropathy [29, 30], however, it is nowadays used for numerous indications like rotator cuff deficiency and proximal humeral fractures. Moreover, the technique has been applied for fracture complications such as malunion or nonunion, chronic dislocations, and revision arthroplasty [10, 11].

The above technique has become a pioneer treatment option for several complex shoulder injuries and disorders of the elderly, providing adequate deltoid elongation and decreasing the forces required to abduct the arm by medializing the center of rotation, overriding the presence of a dysfunctional rotator cuff [31]. In order to improve external rotation of the arm and decrease scapular notching, implants with a lateralized glenosphere have been used [32]. New designs as BIO-RSA (Tornier, Warsaw, USA) support this idea recently. Indications for treating proximal humeral fractures with reverse shoulder arthroplasty are elderly individuals (>70 years) with non-reconstructable tuberosities and patients with high-risk fractures of poor functional potential outcome if treated with ORIF or Hemiarthroplasty (*i.e*. pre- existing rotator cuff tear and arthritis, high likelihood of humeral head osteonecrosis, osteoporotic bone). Contraindications include malfunction of axillary nerve, deltoid dysfunction, scapular or acromion fracture as well as open fractures due to high risk of infection.

### Surgical Approach for Reverse Shoulder Arthroplasty

Reverse shoulder prosthesis utilizes the deltoid function and establishes improved kinetics when there is substantial rotator cuff dysfunction or absence of tuberosities healing. The post-operative goals are improved clinical function and relief of pain [33, 34]. The surgical approach for RSA has been either the standard deltopectoral approach or the anterosuperior approach. Each approach has its benefits and disadvantages. The deltopectoral approach (DP) is the universal and familiar approach to the shoulder. It allows adequate access to the fracture fragments for suture fixation and provides adequate glenoid exposure after fracture fragment mobilization. Also, reducing trauma to the deltoid has a theoretic advantage, given that reverse arthroplasty is powered primarily by the deltoid muscle. However, deltopectoral approach has several disadvantages. The role of the subscapularis though, in function and stability of a reverse arthroplasty is controversial. Several studies have associated subscapularis dysfunction with greater risk of instability as well as increased danger of nerve injury [35-37]. Also, visualization and instrumentation of the posterior glenohumeral structures can be difficult from an anterior approach making the exposure and reduction of the greater tuberosity or the implantation of the base plate a demanding procedure. The anterosuperior approach (AS) uses a more limited superior incision via deltoid split. The deltoid is split between its anterior and middle thirds, starting at the anterolateral corner and extending distally up to 4 cm. The positive outcomes gained when using this technique are the adequate exposure of the glenoid, the better access to the greater tuberosity and the ability to preserve the subscapularis tendon whilst reducing the risk of dislocation. Also, preserving the anterior soft tissue structures provides a compressive effect that may reduce the need to lengthen the arm for stability thus potentially reducing the incidence of neurologic damage or fracture of the acromion [21]. However, the surgeon that will select the AS approach, will probably face difficulties in placing the glenoid baseplate in a neutral or an inferiorly tilted position as well as the exposure humeral shaft due to the limited extensibility of the approach. The requirement for deltoid detachment and potential dehiscence or weakening (damage to the distal branches of axillary nerve) that may lead to postoperative pain or dysfunction. Mole *et al*. [38] performed a multicenter study comparing instability, function and pain scores, scapular notching, and complications in the AS (n = 227) and DP (n = 300) approaches. In 527 reverse arthroplasties with a minimum 2-year follow-up, postoperative instability rate was greater with the DP (5.1%) than with the AS (0.8%) approach (P<.001). Scapular notching occurred in 74% of AS approaches and 63% of DP approaches (P 0 .03). Acromial fractures occurred in 5.6% of DP approaches and 2.2% of AS approaches (P 0 .02).No differences in Constant-Murley score or active mobility were found. When it comes to the treatment of proximal humeral fractures we prefer the deltopectoral approach with the use of reverse shoulder arthroplasty.

### Technical Demands Tuberosity Repair

Although restoring rotator cuff is not obligatory for RSA to be functional for arm elevation, repairing the grater tuberosity and the function of the infraspinatus and teres minor can lead to improved external rotation strength and function. Tuberosity fixation to the implant and shaft remains important for the proximal humeral fractures treatment with RSA. Recent studies have shown that improved rotation may be obtained if the tuberosities are repaired anatomically to the RSA implant [39]. With the increased number of fractures treated with RSA, modern humeral stems offer accelerated healing of tuberosities. Some humeral prosthesis have proximal ingrowth potential to control fragments; others are coated with hydroxyapatite or have windows in the proximal stem for better bone grafting from humeral head to enhance tuberosity integration [10]. Few studies have investigated the role of lesser tuberosity-subscapularis repair with controversial results [40-42]. A deficient subscapularis predisposes the prosthesis to anterior instability, placing additional demands and requiring attention. Moreover, caution is required when repairing the subscapularis, whereby the remaining external rotators may be weakened in the effort to increase the overall internal rotation. Gallinet *et al*. [14] in their study found that patients with anatomic healing of the tuberosities had improved forward elevation, external rotation at the side and at 90^o^ of abduction when compared with patients who had nonunion or malunion of the greater tuberosity. Patients with healed tuberosities also had improved Constant and DASH scores. We believe that tuberosity repair is a necessary procedure for the RSA when used for fractures. We prefer to repair tuberosities with nonabsorbable sutures and careful handling of the tuberosities. The sutures can be placed before glenosphere placement because the implant may limit one’s ability to suture the greater tuberosity. Additionally, vertical sutures are also placed to secure the shaft to the tuberosities in order to avoid proximal migration of the tuberosities to the implant.

### Implant Positioning and Soft Tissue Tension

RSA longevity and impingement-free range of motion depend on implant positioning and prosthetic design. The baseplate should be positioned inferiorly on the glenoid to limit scapular notching. In order to achieve that, inferior capsular release is essential for glenoid exposure for proper baseplate positioning. The three -column concept has been introduced for fixation and positioning of the glenoid baseplate [43]. Newer studies support the perpendicular position of the lower screw in order to avoid complications after notching and support superior fixation [44]. One screw is aimed at the coracoid, one toward the scapular spine, and one down the lateral pillar. Recent studies have shown that the glenoid baseplate may only need 2 screws for adequate fixation. Glenoid component positioning is an important factor in total shoulder arthroplasty. Preoperative planning should include CT with three-dimensional (3D) reconstruction for better imaging of glenoid retroversion, inclination, and humeral head subluxation [45, 46]. Inferior tilting of the glenosphere has been proposed from the majority of the published studies although this position may lead to earlier contact of the humerus with the lateral scapula [47, 48]. Increased varus humeral neck-shaft angle has been correlated with less adduction deficits and scapular notching [49]. Rotation of the prosthesis affects range of motion, stability of the arthroplasty and tuberosity healing. Less humeral retroversion leads to decreased external rotation before impingement [31]. We prefer 20^o^ retroversion with regard to the forearm of the patient. Adequate soft tissue tensioning is an important factor for the stability and proper deltoid function that leads to better outcomes. Both horizontal and axial tension is important to consider. Lengthening of the arm up to 1.5 cm leads to sufficient deltoid tension and improved forward flexion [50, 51]. However, the humerus should not be lengthened more than 2.5 cm compared with the opposite side because overlengthening has been correlated with neurologic complications, deltoid fatigue and stress fracture of the acromion [10, 31].

## RESULTS OF RSA IN FRACTURES

**Fig. (4):**
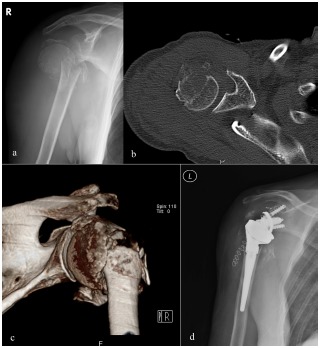
X-Ray (**a**), CT (**b**) and 3D reconstruction CT (**c**) of a 4-part humeral head fracture treated with reverse shoulder arthroplasty.

Recent studies have shown promising results for RSA in proximal humeral fractures treatment (Fig. **4**). Klein *et al*. [52] investigated 20 patients with a mean follow up of 33 months and found improvement of Constant and SF 36 scores close to age-related norms when the Delta III RSA (Depuy Orthopaedics, Warsaw, IN) was used for acute fractures in the elderly. Bufquin *et al*. [39] prospectively evaluated the use of RSA for the treatment of 3- and 4-part fractures in elderly with a mean follow up of 22 months.

He concluded that tuberosity nonunion may not pose a threat to the final outcome and that the RSA technique/method is the recommended option for patients suffering both comminuted tuberosities and osteoporosis. Although these initial reports have shown good results, further studies have disclosed the high incidence of scapular notching, neurologic complications, and component loosening seen at midterm follow-up using the RSA for the treatment of fractures. According to previous studies, RSA in patients for proximal humeral fractures has shown better outcomes at short and midterm follow-up with forward flexion improvement, compared to hemiarthroplasty [53, 54]. Limitations of these studies are retrospective design, small population with short follow-up period and heterogeneity of the results [55]. Finally RSA can also be used as a salvage procedure after failure of internal fixation of proximal humeral fractures with a recently published study showing 79% of the patients with excellent outcomes [56].

### COMPLICATIONS

**Fig. (5):**
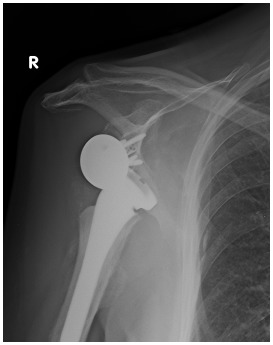
Dislocation of a reverse shoulder arthroplasty.

Complication rates in the literature have ranged from 0% to 68% [57] including complex regional pain syndrome, wound infection, scapular notching, hematoma, nerve injuries, instability, early loosening and periprosthetic fracture. The procedure has a steep learning curve; however, the complication rate has shown decrease after 40 cases [58]. Scapular notching is a radiographic finding due to a reaction to prosthesis material (polyethylene) with high incidence (up to 96%) especially with Grammont-style prosthesis [59, 60]. Reaction begins with contact between the scapular neck and the polyethylene during adduction and can result to baseplate fixation, poor outcomes and prosthesis failure [59]. It has been correlated with superior positioning of the glenosphere, medialization of the center of rotation and high body mass. Instability is common after reverse prosthesis with rates of dislocation reported as high as 30% [4, 61] (Fig. **5**). Post -operative complications may be the result of either soft tissue tension, glenosphere diameter, or humeral- socket constraint, impingement, and deltoid dysfunction. According to Gutierrez *et al*. [62] a number of factors of prosthetic design, including the compressive force on the prosthesis, the prosthetic socket depth, and the glenosphere size may be related to dislocation. According to their study, increasing the compressive forces on the prosthesis followed by humerosocket depth lead to improved stability. Furthermore, stability of the reverse shoulder arthroplasty may be achieved by lengthening the humerus. Yet, increasing humeral length poses a threat for deltoid over-tensioning and loss of motion, acromion stress fracture and brachial plexopathy [51]. Infection of a reverse shoulder arthroplasty has been reported as high as 4% and could be a catastrophic with difficult management complication [4]. The most common micro-organism found are Propionibacterium acnes and Staphylococcus epidermidis [63, 64].

## CONCLUSION

Comminuted proximal humerus fractures in the elderly may present a number of drawbacks. RSA technique has become a successful tool for dealing with some of these drawbacks.

Careful preoperative planning and attention in tuberosity fixation are important factors in order to achieve good clinical outcomes. Short-term results of comparative studies between RSA and Hemiarthroplasty showed improved forward flexion, abduction and Constant scores in patients treated with RSA. Long term follow up studies as well as randomized studies are needed to be done in order to give safe conclusions for the use of RSA in proximal humeral fractures.

## LIST OF ABBREVIATIONS

AS: = Anterosuperior approach
DP: = Deltopectoral approach
RSA: = Reverse shoulder arthroplasty
